# Supplementary material for: Characterization of Bifidobacterium asteroides Isolates
Source: Microorganisms. 2022 Mar 18;10(3):655. doi: 10.3390/microorganisms10030655 (PMC8950671; doi:10.3390/microorganisms10030655)
Supplement: Supplementary file 1 [file microorganisms-10-00655-s001.zip › microorganisms-1610360-supplementary.pdf]

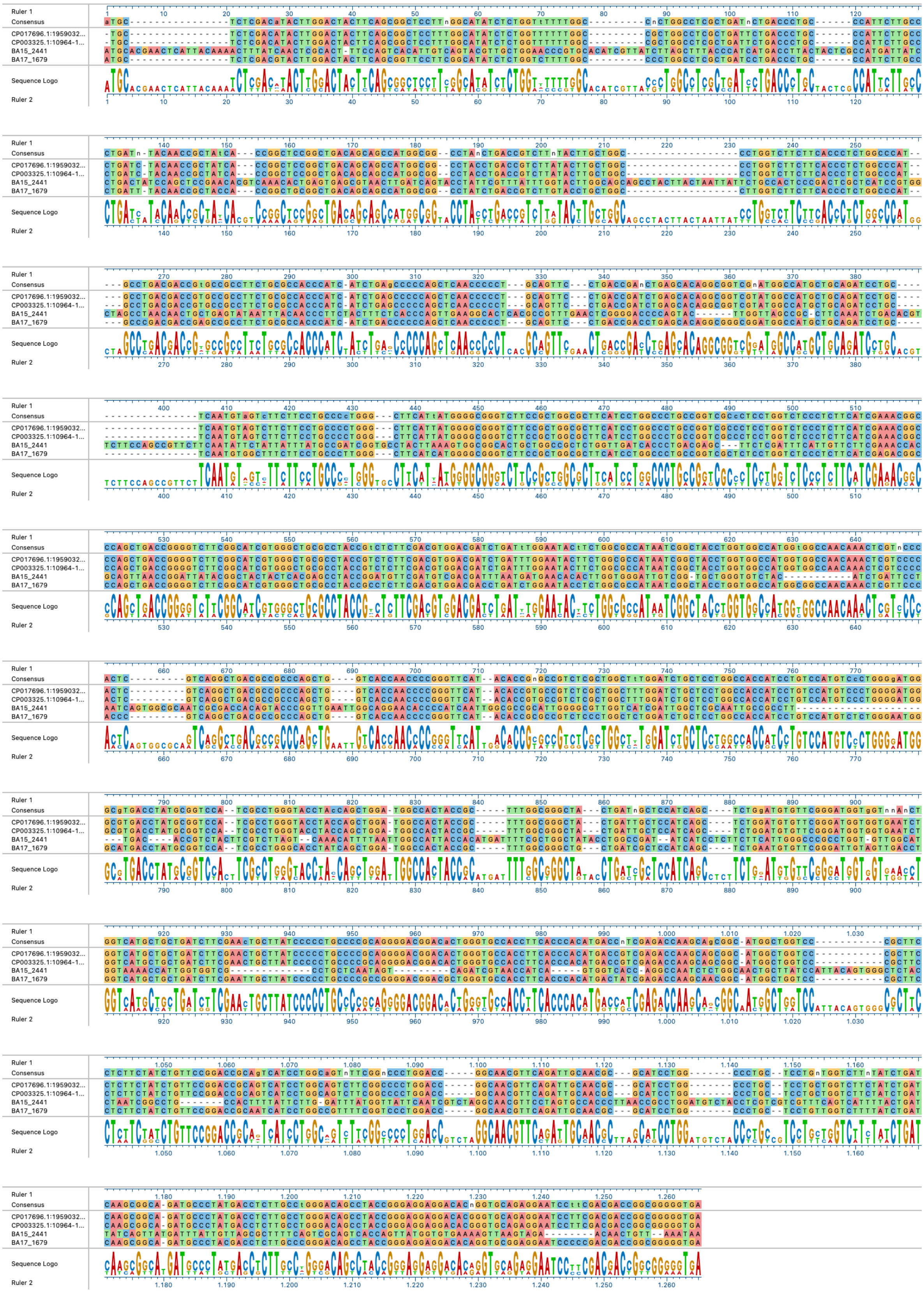

Figure S1. Nucleotide sequences of the putative *vanZ* gene of BA15 and BA17 strains and their distance with the DSM 20089 and PRL2011 strains aligned using MUSCLE.
